# Ethylene/Styrene Copolymerization by (Me_3_SiC_5_H_4_)TiCl_2_(O-2,6-*^i^*Pr_2_-4-RC_6_H_2_) (R = H, SiEt_3_)-MAO Catalysts: Effect of SiMe_3_ Group on Cp for Efficient Styrene Incorporation

**DOI:** 10.3390/molecules29184473

**Published:** 2024-09-20

**Authors:** Tiantian Huang, Taiga Fujioka, Daisuke Shimoyama, Kotohiro Nomura

**Affiliations:** Department of Chemistry, Tokyo Metropolitan University, 1-1 Minami Osawa, Hachioji 192-0397, Tokyo, Japandshimo@tmu.ac.jp (D.S.)

**Keywords:** titanium, catalyst, homogeneous catalyst, copolymerization, half-titanocene, ethylene, styrene, ligand effect, cyclopentadienyl

## Abstract

The synthesis and structural analysis of (Me_3_SiC_5_H_4_)TiCl_2_(OAr) [OAr = O-2,6-*^i^*Pr_2_-4-RC_6_H_2_; R = H, SiEt_3_] revealed that it exhibits higher catalytic activities than (*^t^*BuC_5_H_4_)TiCl_2_(OAr), Cp*TiCl_2_(OAr), with efficient comonomer incorporation in ethylene/styrene copolymerization in the presence of a methylaluminoxane (MAO) cocatalyst. The catalytic activity in the copolymerization increased upon increasing the charged styrene concentration along with the increase in the styrene content in the copolymers, whereas the activities of other catalysts showed the opposite trend. (Me_3_SiC_5_H_4_)TiCl_2_(O-2,6-*^i^*Pr_2_C_6_H_3_) displayed the most suitable catalyst performance in terms of its activity and styrene incorporation, affording amorphous copolymers with styrene contents higher than 50 mol% (up to 63.6 mol%) and with random styrene incorporation confirmed by ^13^C-NMR spectra.

## 1. Introduction

Olefin coordination polymerization using transition metal catalysts is the core technology for the production of polyolefins, and the development of new polymeric materials, especially copolymers consisting of ethylene or propylene with monomers which have never been used by conventional catalysts (Ziegler–Natta, metallocene catalysts, etc.), is a long-term interest in this research field. Catalyst development has been considered to play an important key role in the success of this research area [1,2,3,4,5,6,7,8,9,10]. Ethylene copolymers with aromatic vinyl monomers exemplified as ethylene/styrene copolymers are known to be interesting materials whose thermo-mechanical and viscoelastic properties can be modified through the incorporation of a comonomer (styrene) [11]. Half-titanocene complexes [5,6,7,8,9,11,12,13,14,15,16,17,18,19,20,21,22,23,24,25,26,27,28] have been known as efficient catalysts in terms of having a better capacity for styrene incorporation than ordinary metallocene catalysts [11,29,30].

**Scheme 1:**
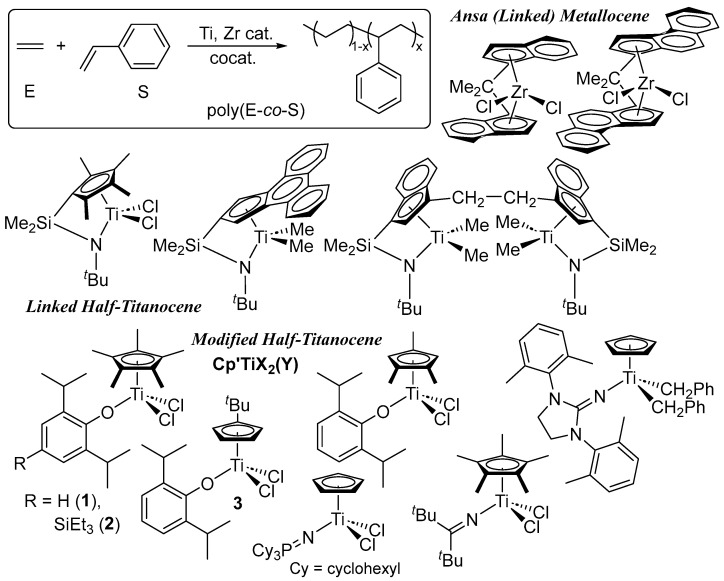
Selected titanium and zirconium catalysts for ethylene/styrene copolymerization.

Linked (*ansa*) half-titanocenes (called “constrained geometry type”) [5,6,7,12,13,14,15,16,17,18,19], certain *ansa*-metallocenes [29,30], and non-bridged modified half-titanocenes (exemplified in Scheme 1) [20,21,22,23,24,25,26,27,28] are known to be effective transition metal catalysts in ethylene/styrene copolymerization [11]. In particular, the bimetallic linked half-titanocene, (CH_2_CH_2_)[Me_2_Si(indenyl)(N*^t^*Bu)TiMe_2_]_2_ [17,19], and the phenoxide-modified half-titanocene, Cp’TiCl_2_(O-2,6-*^i^*Pr_2_C_6_H_3_) [Cp’ = *^t^*BuC_5_H_4_ (**3**), 1,2,4-Me_3_C_5_H_2_] [20,21], have been recognized as effective catalysts in terms of the synthesis of copolymers with high styrene contents (>50 mol%) [17,19,20,21]. The resultant copolymers with high styrene contents (ca. >30 mol%) are amorphous, and the glass transition temperature (*T*_g_) increases with the increase in the styrene content (shown below) [21]. However, as described above, catalysts affording copolymers with high styrene contents (especially >50 mol%) are still limited. Moreover, these phenoxide-modified catalysts (shown above) also exhibited remarkable catalytic activities for syndio-specific styrene polymerization [20,31], whereas the bimetallic linked half-titanocene gave atactic polystyrene [17]. In contrast, the C_5_Me_5_ (Cp*) analogues, Cp*TiCl_2_(O-2,6-*^i^*Pr_2_-4-RC_6_H_2_) [R = H (**1**), SiEt_3_ (**2**), Scheme 1], exhibited remarkable catalytic activities for ethylene copolymerization with α-olefins [3,8,9]; the SiEt_3_ analogue (**2**) was effective for ethylene copolymerization with alken-1-ol [32]. These results also suggest that modification of the cyclopentadienyl fragment plays a role in the success of efficient catalysts for the desired ethylene copolymerization [3,8,9]; an introduction of the SiEt_3_ group would lead to higher activity as a result of better π-donation [32].

**Scheme 2:**
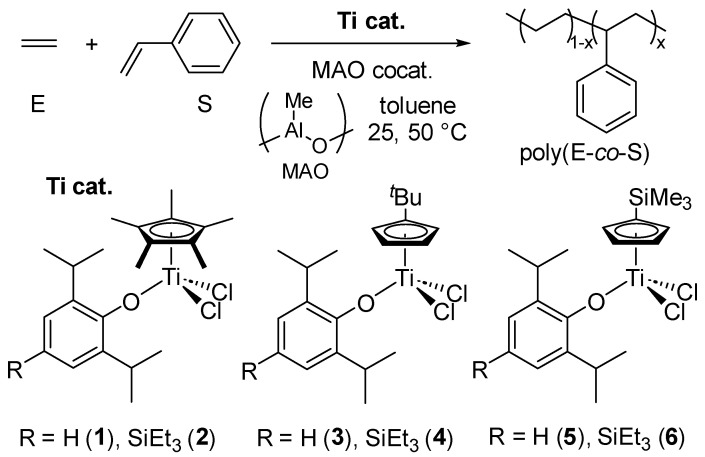
Ethylene/styrene copolymerization by phenoxide−modified half−titanocene catalysts.

In this paper, we focus on the phenoxide−modified half−titanocene catalysts for ethylene/styrene copolymerization. We recently realized that the complexes containing the trimethylsilyl-cyclopentadienyl ligand, (Me_3_SiC_5_H_4_)TiCl_2_(O-2,6-*^i^*Pr_2_C_6_H_3_), showed better catalyst performance than the *tert*-BuC_5_H_4_ analogue (**3**) [20,21] in the copolymerization. We thus herein report our results concerning the synthesis of (Me_3_SiC_5_H_4_)TiCl_2_(O-2,6-*^i^*Pr_2_-4-RC_6_H_2_) [R = H (**5**), SiEt_3_ (**6**)] and its use as a catalyst for ethylene/styrene copolymerization in the presence of a methylaluminoxane (MAO) cocatalyst (Scheme 2).

## 2. Results and Discussion

### 2.1. Synthesis and Structural Analysis of (Me_3_SiC_5_H_4_)TiCl_2_(O-2,6-^i^Pr_2_-4-RC_6_H_2_) (R = H, SiEt_3_)

**Figure 1:**
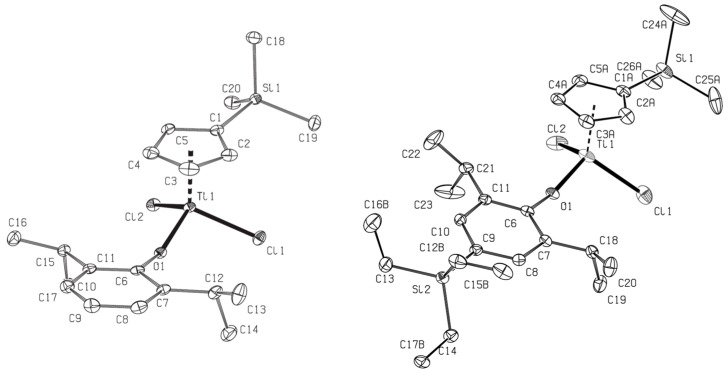
ORTEP drawings for (Me_3_SiC_5_H_4_)TiCl_2_(O-2,6-*^i^*Pr_2_-4-RC_6_H_2_) [R = H (**5**, **left**), SiEt_3_ (**6**, **right**)]. Thermal ellipsoids are drawn at 30 % probability level, and hydrogen atoms are omitted for clarity. Additional data including crystal data and collection parameters for structural analysis are shown in Supplementary Material.

The phenoxide-modified half-titanocenes containing the Me_3_SiC_5_H_4_ ligand, (Me_3_SiC_5_H_4_)TiCl_2_(O-2,6-*^i^*Pr_2_-4-RC_6_H_2_) [R = H (**5**), SiEt_3_ (**6**)] were prepared according to the previous report by treating (Me_3_SiC_5_H_4_)TiCl_3_ [33,34] with Li(O-2,6-*^i^*Pr_2_C_6_H_3_) or 2,6-*^i^*Pr_2_-4-SiEt_3_-C_6_H_2_OH (in the presence of NEt_3_) in diethyl ether. (*^t^*BuC_5_H_4_)TiCl_2_(O-2,6-*^i^*Pr_2_-4-SiEt_3_-C_6_H_2_) (**4**) was also prepared in the same manner (as described in the Experimental Procedures Section). These complexes were identified by NMR spectra and elemental analysis, and the structures of **5** and **6** were determined by X-ray crystallography (Figure 1, CCDC 2378274, 2378275; the table for the crystal data and the collection parameters are shown in the Supplementary Material). The selected bond distances and angles are summarized in Table 1. The data for Cp*TiCl_2_(O-2,6-*^i^*Pr_2_-4-RC_6_H_2_) [R = H (**1**) [35], SiEt_3_ (**2**) [32]] and CpTiCl_2_(O-2,6-*^i^*Pr_2_C_6_H_3_) (**7**) [35] are inserted for comparison.

The analyses revealed that these complexes fold a distorted tetrahedral geometry around titanium, and no significant differences are observed in Ti–Cl, Ti–O, and O–C (phenyl) bond distances among these complexes. It seems that Ti–C(1) and Ti–C(2) in **5** [2.3678(17), 2.3854(19) Å] and Ti–C(4) and Ti–C(5) in **6** [2.390(6), 2.398(8) Å] are rather longer than the others in the cyclopentadienyl ligands, and these results might lead to an assumption that the cyclopentadienyl group would be coordinated as *η*^3^-fashion. However, these phenomena were also seen in complexes **1** [2.438(7) Å in Ti–C(2)] [35], **2** [2.403(3), 2.411(3) Å in Ti–C(1) and Ti–C(2)] [32], and even **7** [2.299(5) Å in Ti–C(2) and Ti–C(5)] [35]. Therefore, it seems more likely that the observed fact could be probably due to crystal packing in the crystallographic analysis. It was revealed that the Ti(1)–O(1)–C(6 in phenyl) angles in **5** [151.41(12)°] and **6** [157.0(2)°] are apparently smaller than those in **1** and **2** [173.0(3), 174.62(19)°, respectively] [32,35] and **7** [163.0(4)°] [35], suggesting that these complexes (**5**,**6**) show an inferior π-donation capability from the phenoxide ligand.

### 2.2. Ethylene/Styrene Copolymerization by Cp′TiCl_2_(O-2,6-^i^Pr_2_-4-RC_6_H_2_) [Cp’ = Cp*, ^t^BuC_5_H_4_, Me_3_SiC_5_H_4_, R = H, SiEt_3_]–MAO Catalyst Systems

Table 2 summarizes the results of the ethylene/styrene copolymerization using a series of phenoxide-modified half-titanocene catalysts, Cp′TiCl_2_(O-2,6-*^i^*Pr_2_-4-RC_6_H_2_) [Cp’ = Cp*, R = H (**1**), SiEt_3_ (**2**); Cp’ = *^t^*BuC_5_H_4_, R = H (**3**), SiEt_3_ (**4**); Cp’ = Me_3_SiC_5_H_4_, R = H (**5**), SiEt_3_ (**6**)] in the presence of an MAO cocatalyst. MAO was used as the white solid (d-MAO) after the removal of AlMe_3_ and toluene from the commercially available samples [TMAO-S, 9.5 wt% (Al) toluene solution, Tosoh Finechem Co., Shunan, Japan], because the use of MAO is quite effective for obtaining high-molar-mass polymers efficiently, as shown in the previous reports [8,9,20,21,28,32,35]. The resultant polymers usually contained atactic polystyrene generated by MAO itself, and the polymer yields were described after the removal of atactic polystyrene through extraction with acetone (see Experimental Procedures). As shown in Table 2, the contents (atactic polystyrene) were negligible in most cases (>99%), except when the copolymerization runs were conducted by the Cp* analogues (**1**,**2**) under high initial styrene concentrations (runs 2–6) due to low catalytic activities (low copolymer yields). These copolymerization runs were terminated after 10 min to control the comonomer (styrene) conversion so it was less than 10% in order to obtain copolymers with uniform compositions. For instance, the styrene conversions in runs 7–15 are 10%, 6.4%, 3.2%, 4.6%, 2.8%, 1.3%, 7.9%, 7.2%, and 6.5%, respectively.

As reported previously [20,21], the *tert*-BuC_5_H_4_ analogue (**3**) showed higher catalytic activities than the Cp* analogue (**1**), and the activity based on the polymer yield decreased with the increase in the initial charged styrene concentration along with the decrease in the *M*_n_ values (runs 1–3 vs. runs 7–9). The resultant copolymers were amorphous and possessed uniform compositions, confirmed by DSC thermograms [observed as a sole glass transition temperature (*T*_g_)]; their uniform compositions were also confirmed previously by GPC–FT-IR and cross-fractionation (CFC) analyses [21]. The *T*_g_ value increased with the increasing styrene content (estimated by ^1^H NMR spectra according to the reported procedure) [20,21]. It was revealed that the introduction of the SiEt_3_ group into the *para*-position in the phenoxide led to a decrease in the catalytic activities. The activities of **2** and **4** were lower than those of **1** and **3**, respectively, whereas no remarkable differences were observed in the molecular weight and the styrene contents in the copolymers. There was an opposite trend in the ethylene copolymerization with α-olefin, 2-methyl-1-pentene reported previously for **1** and **2** [32], although we are not sure of the reason for this at this moment. As shown in Table S2 (Supplementary Materials), as reported previously for **1** and **3**,^21^ the catalytic activities based on the polymer yields decreased upon the addition of styrene.

**Figure 2:**
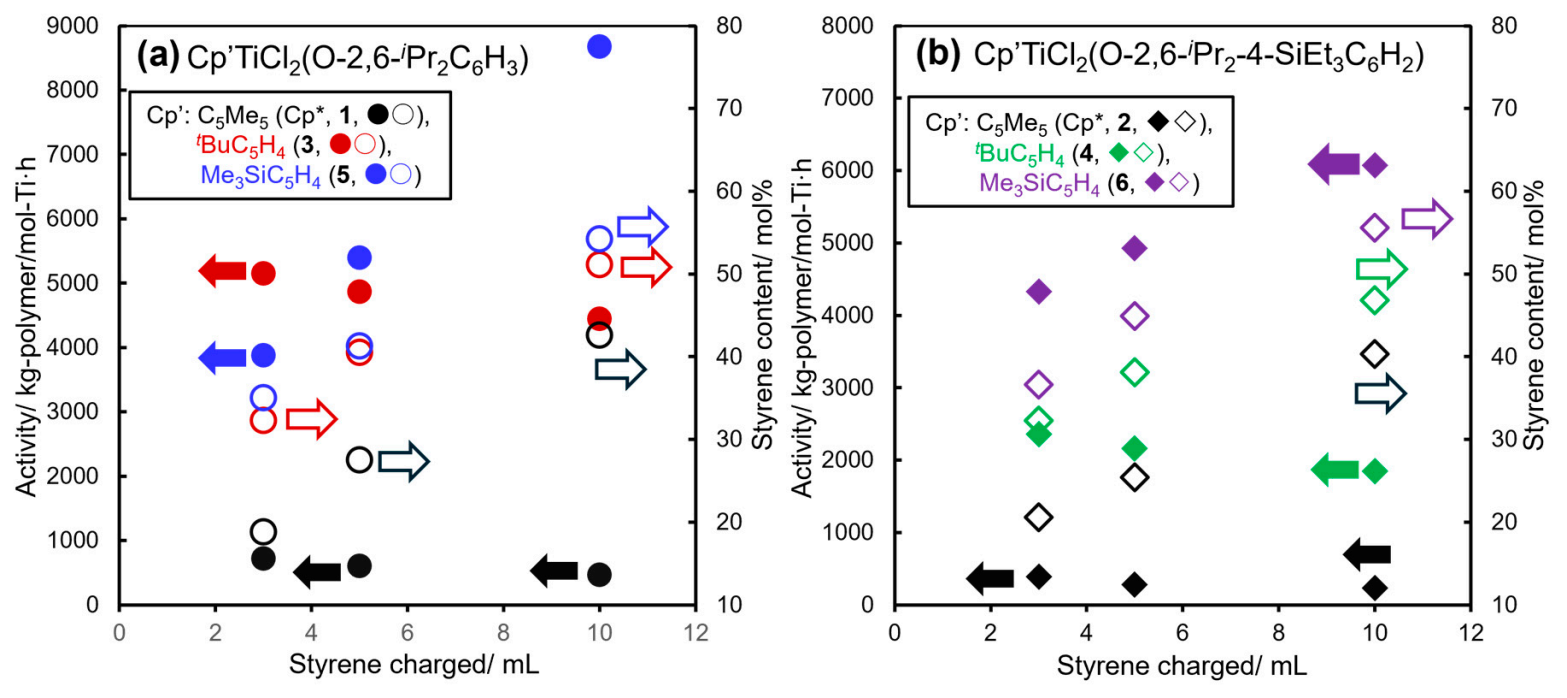
The effect of the cyclopentadienyl fragment on the catalytic activity and the styrene contents of the copolymers in ethylene/styrene copolymerization by Cp′TiCl_2_(O-2,6-*^i^*Pr_2_-4-RC_6_H_2_) [Cp’ = Cp*, R = H (**1**), SiEt_3_ (**2**); Cp’ = *^t^*BuC_5_H_4_, R = H (**3**), SiEt_3_ (**4**); Cp’ = Me_3_SiC_5_H_4_, R = H (**5**), SiEt_3_ (**6**)]–MAO catalyst systems. The detailed data are shown in Table 2. The filled circle or square (arrows) expresses activity and the others express the styrene contents.

It should be noted that the Me_3_SiC_5_H_4_ analogue (**5**) showed higher catalytic activity than the others (**1**–**4**), and the styrene contents in the copolymers were close to those produced by the *tert*-BuC_5_H_4_ analogue (**3**) under the same conditions (Table 2, Figure 2). Note that the activity by **5** increased upon increasing the charged styrene concentration along with the increase in the styrene content in the resultant copolymers (runs 13–15, Figure 2); the *M*_n_ value in the resultant copolymer decreased with the increase in the styrene content. Similarly to the observed trend for **2** and **4**, the introduction of the SiEt_3_ group into the *para*-position in the phenoxide (**6**) led to a decrease in the catalytic activities (runs 16–18). As observed in the copolymerization by **5**, the activity of **6** also increased upon increasing the charged styrene concentration along with the increase in the styrene content in the copolymers. It seems that the *M*_n_ values in the copolymers prepared with **6** were higher than those prepared with **5**.

**Figure 3:**
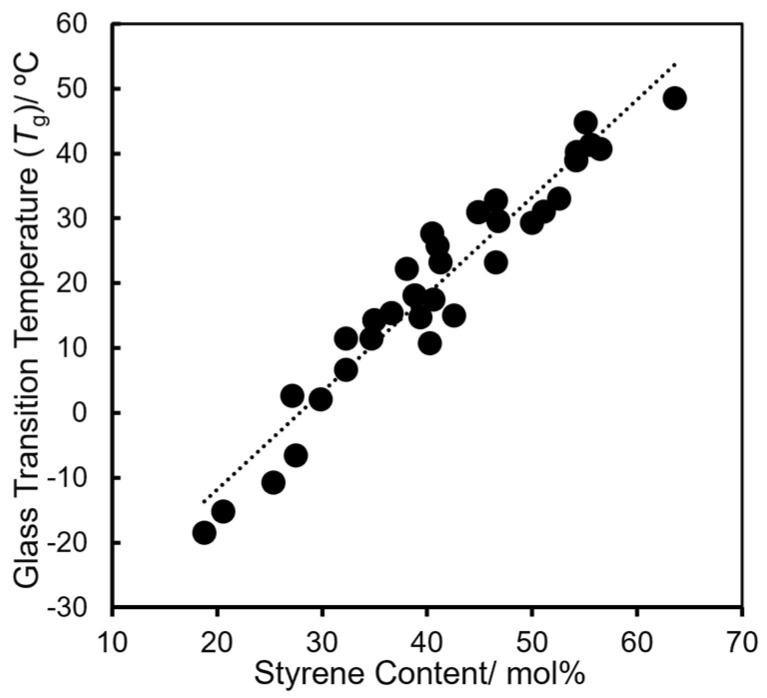
Plots of the glass transition temperature (*T*_g_/°C) versus the styrene content (mol%) in the copolymers estimated by ^1^H NMR spectra (in 1,1,2,2−tetrachloroethane-*d_2_* at 110 °C). The plots were made on the basis of the copolymerization data by catalysts **1**–**6** in the presence of MAO cocatalyst.

Table 3 summarizes the results of the ethylene/styrene copolymerization by **5** and **6** under various conditions (temperature, ethylene pressure). The activity of **5** was affected by the ethylene pressure employed (runs 13, 19, and 28) and increased upon increasing the ethylene pressure along with the decrease in the styrene content in the resultant copolymer. A decrease in the catalyst concentration (0.50 → 0.30 μmol) did not affect the activity, the *M*_n_ values, and the styrene content in the copolymers (runs 13–15, 20–22). The styrene content in the copolymers reached to 63.6 mol% when the copolymerization was conducted under high styrene and low ethylene concentration conditions (run 29). Unfortunately, the activities conducted at 50 °C (runs 23–27) were low compared to those conducted at 20 °C (runs 13–15), and the resultant copolymers possessed low *M*_n_ values compared to those conducted at 20 °C. A similar trend was also observed in the copolymerization using the **6**–MAO catalyst system (runs 30–33 vs. runs 16–18), whereas the *M*_n_ values in the copolymers were higher than those prepared with **5** under the same conditions. As shown in Figure 3, a linear correlation between the *T*_g_ values with the styrene contents in the copolymers was observed, as seen in a previous report [11].

**Figure 4:**
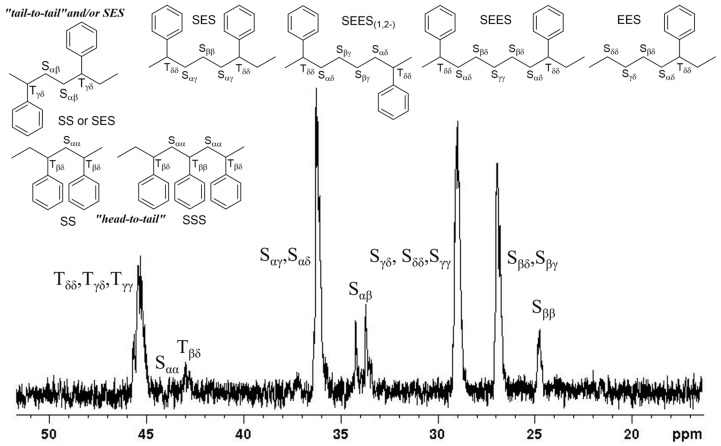
^13^C NMR spectrum (in C_2_D_2_Cl_4_ at 110 °C, methylene and methine region) of poly(ethylene-*co*-styrene) prepared by (Me_3_SiC_5_H_4_)TiCl_2_(O-2,6-*^i^*Pr_2_C_6_H_3_) (**5**)–MAO catalyst system; styrene content of 35.0 mol% (run 13).

Figure 4 shows a typical ^13^C NMR spectrum (in 1,1,2,2-tetrachloroethane-*d_2_* at 110 °C) in the ethylene/styrene copolymer prepared by the **5**–MAO catalyst system (run 13, styrene 35.0 mol%). As reported previously for catalysts **1** and **3** [11,20,21], the resonances corresponding to styrene head-to-tail incorporation and SES or SS (S_ββ_, tail-to tail, called pseudo random) were observed in addition to the resonances ascribed to isolated and alternating styrene incorporation (depicted in Figure 4). These results clearly suggest that styrene incorporation was random in this catalysis. No significant differences in the spectra were observed in the resultant copolymers prepared with **6** (the additional data are shown in the Supplementary Material).

## 3. Conclusions

We have demonstrated that newly prepared (Me_3_SiC_5_H_4_)TiCl_2_(OAr) [OAr = O-2,6-*^i^*Pr_2_-4-RC_6_H_2_; R = H (**5**), SiEt_3_ (**6**)] exhibited higher catalytic activities than (*^t^*BuC_5_H_4_)TiCl_2_(OAr), Cp*TiCl_2_(OAr), with efficient comonomer incorporation, in ethylene/styrene copolymerization in the presence of an MAO cocatalyst at 20 ºC. The catalytic activity in the copolymerization increased upon increasing the charged styrene concentration along with the increase in the styrene content in the copolymers, whereas the activities of other catalysts showed the opposite trend. Catalyst **5** showed the most suitable catalyst performance in terms of its activity and styrene incorporation, affording amorphous copolymers containing styrene contents higher than 50 mol% (up to 63.6 mol%) with random styrene incorporation. The unique characteristics observed in **5** should be helpful for further designing efficient molecular catalysts for the synthesis of new polyolefins and should provide a new idea for the metal-catalyzed polymerization of various monomers.

## 4. Materials and Methods

General. All experiments were conducted under a nitrogen atmosphere unless otherwise specified. Anhydrous-grade toluene (Kanto Chemical Co., Inc., Tokyo, Japan) containing a mixture of molecular sieves (3 Å 1/16 and 4 Å 1/8, and 13 × 1/16) was stored in a dry box, and styrene (Tokyo Chemical Industry Co., Ltd., Tokyo, Japan) was stored under nitrogen in a freezer in the dry box and passed through an alumina short column before use. Ethylene (polymerization grade, purity > 99.9%; Sumitomo Seika Co., Ltd., Osaka, Japan) was used as received. According to previous reports [20,21,28,32,35], toluene and AlMe_3_ in the commercially available MAO solution [TMAO-S, 9.5 wt% (Al) toluene solution, Tosoh Finechem Co.] were removed under reduced pressure (at ca. 50 °C and completion for 1 h at >100 °C) to afford an AlMe_3_-free MAO white solid (d-MAO) as the cocatalyst employed in this study. Cp′TiCl_2_(O-2,6-*^i^*Pr_2_-C_6_H_3_) [Cp′=Cp* (**1**), *^t^*BuC_5_H_4_ (**3**)] [35] and Cp*TiCl_2_(O-2,6-*^i^*Pr_2_-4-SiEt_3_-C_6_H_2_) (**2**) [32] were prepared according to previous reports. TMAO-S generates flammable gases upon exposure to water and/or air, and should be kept under an inert atmosphere.

All ^1^H and ^13^C NMR spectra were recorded on a Bruker AV500 spectrometer (Bruker 500.13 MHz for ^1^H, 125.77 MHz for ^13^C, Billerica, MA, USA), and chemical shifts are given in ppm and are referenced to SiMe_4_. All spectra for the analysis of the titanium complexes were obtained in the solvent indicated at 25 °C, and the resultant poly(ethylene-*co*-styrene)s were prepared by dissolving them in 1,1,2,2-tetrachloroethane-*d*_2_ and were measured at 110 °C. ^13^C{^1^H} NMR spectra (90° pulse angle) were measured with the conditions of a pulse interval of 5.2 s and an acquisition time of 0.8 s; number of the transients in the analysis was accumulated, ca. 6000. Gel permeation chromatography (GPC) was performed to estimate the molecular weights (*M*_w_, *M*_n_) and their distributions, and the analysis was conducted by using Tosoh HLC-8321GPC/HT (at 140 °C) in *ortho*-dichlorobenzene (containing 0.05 wt% 2,6-di-*tert*-butyl-*p*-cresol) as the eluent. The molecular weight was estimated with a calibration curve by using polystyrene standard samples. Thermal properties in the resultant poly(ethylene-*co*-styrene)s were analyzed by differential scanning calorimetric (DSC) thermograms (Hitachi DSC-7000X, Hitachi High-Tech Corp. Tokyo, Japan) under nitrogen atmosphere. The analysis of the copolymer samples was conducted upon heating from −100 to 250 °C at 20 °C/min, after preheating from 30 to 250 °C (20 °C/min) and cooling to −100 °C (10 °C/min). In this heating scan, the melting temperature (*T*_m_) and the glass transition temperature (*T*_g_) were chosen from the middle of the phase transition.

Preparation of (*^t^*BuC_5_H_4_)TiCl_2_(O-2,6-*^i^*Pr_2_-4-SiEt_3_C_6_H_2_) (**4**). An Et_2_O solution (5.0 mL) containing 2,6-*^i^*Pr_2_-4-SiEt_3_-C_6_H**_2_**OH (292 mg, 1.0 mmol) and Et_3_N (152 mg, 1.5 mmol) was added into an Et_2_O (20 mL) solution of (*^t^*BuC_5_H_4_)TiCl_3_ (233 mg, 0.85 mmol) in one portion at −30 °C. The reaction mixture was warmed slowly to room temperature and was stirred overnight. The mixture was then filtered through a Celite pad, and the filter cake was washed with Et_2_O. The combined filtrate and the wash were taken to dryness under reduced pressure to give an orange oil. The resultant oil was then dissolved in a small amount of *n*-hexane. The chilled (−30 °C) solution in the freezer gave orange microcrystals (yield: 76%). ^1^H NMR (500 MHz, CDCl_3_): δ = 7.20 (s, 2H, Ar-*H*), δ = 6.82 (t, 2H, *J* = 2.8 Hz, Cp-*H*), δ = 6.23 (t, 2H, *J* = 2.8 Hz, Cp-*H*), δ = 3.30–3.22 (m, 2H, Ar-C*H*(CH_3_)_2_), δ = 1.44 (s, 9H, Cp-C(C*H*_3_)_3_), δ = 1.23 (d, 12H, *J* = 6.9 Hz, Ar-CH(C*H*_3_)_2_), δ = 0.99 (t, 9H, *J* = 7.9 Hz, Ar-SiCH_2_C*H*_3_), δ = 0.78 (q, 6H, *J* = 7.9 Hz, Ar-SiC*H*_2_CH_3_). ^13^C{^1^H} NMR (125 MHz, CDCl_3_): δ = 165.4, 151.4, 137.2, 133.3, 129.3, 119.3 (Aromatic group), δ = 34.2 (Cp-*C*(CH_3_)_3_), δ = 31.0 (Cp-C(*C*H_3_)_3_), δ = 27.0 (Ar-*C*H(CH_3_)_2_), δ = 23.9 (Ar-CH(*C*H_3_)_2_), δ = 7.66 (-SiCH_2_*C*H_3_), δ = 3.73 (-Si*C*H_2_CH_3_).

Preparation of (Me_3_SiC_5_H_4_)TiCl_2_(O-2,6-*^i^*Pr_2_-C_6_H_3_) (**5**). Li(O-2,6-*^i^*Pr_2_C_6_H**_3_**) (221 mg, 1.2 mmol) was added in one portion to an Et_2_O solution (30 mL) containing (Me_3_SiC_5_H_4_)TiCl_3_ (350 mg, 1.2 mmol) precooled at −30 °C. The reaction mixture was warmed slowly to room temperature and was stirred overnight. The mixture was then filtered through a Celite pad, and the filter cake was washed with Et_2_O. The combined filtrate and the wash were taken to dryness under reduced pressure to give orange solids. The resultant solids were then dissolved in a minimum amount of dichloromethane layered by a small amount of *n*-hexane. The chilled (−30 °C) solution in the freezer gave orange microcrystals (yield: 82%). ^1^H NMR (500 MHz, CDCl_3_): δ = (d, 2H, *J* = 7.8 Hz, Ar-*H*), δ = (t, 1H, *J* = 7.2 Hz, Ar-*H*), δ = 7.04 (t, 2H, *J* = 1.9 Hz, Cp-*H*), δ = 6.40 (t, 2H, *J* = 2.2 Hz, Cp-*H*), δ = 3.27–3.19 (m, 2H, Ar-C*H*(CH_3_)_2_), δ = 1.23 (d, 12H, *J* = 6.8 Hz, Ar-CH(C*H*_3_)_2_), δ = 0.40 (s, 9H, Cp-Si(C*H*_3_)_3_). ^13^C{^1^H} NMR (125 MHz, CDCl_3_): δ = 164.7, 138.3, 136.9, 127.9, 124.5, 123.5, 122.6 (Aromatic group), δ = 27.1 (Ar-*C*H(CH_3_)_2_), δ = 23.7 (Ar-CH(*C*H_3_)_2_), δ = −0.43 (-Si(*C*H_3_)_3_). EA: Found. C 55.52%, H 7.02%; Calcd. for C_20_H_30_Cl_2_OSiTi: C 55.44%, H 6.98%.

Preparation of (Me_3_SiC_5_H_4_)TiCl_2_(O-2,6-*^i^*Pr_2_-4-SiEt_3_C_6_H_2_) (**6**). An Et_2_O solution (5.0 mL) containing 2,6-*^i^*Pr_2_-4-SiEt_3_-C_6_H**_2_**OH (292 mg, 1.0 mmol) and Et_3_N (152 mg, 1.5 mmol) was added in one portion into an Et_2_O solution (20 mL) containing (Me_3_SiC_5_H_4_)TiCl_3_ (262 mg, 0.9 mmol) at −30 °C. The reaction mixture was warmed slowly to room temperature and was stirred overnight. The mixture was then filtered through a Celite pad, and the filter cake was washed with Et_2_O. The combined filtrate and the wash were taken to dryness under reduced pressure to give an orange oil. The oil was then dissolved in a small amount of *n*-hexane, and the chilled (−30 °C) solution in the freezer gave orange microcrystals (yield: 75%). ^1^H NMR (500 MHz, CDCl_3_): δ = 7.20 (s, 2H, Ar-*H*), δ = 7.04 (t, 2H, *J* = 2.3 Hz, Cp-*H*), δ = 6.43 (t, 2H, *J* = 2.3 Hz, Cp-*H*), δ = 3.27–3.19 (m, 2H, Ar-C*H*(CH_3_)_2_), δ = 1.23 (d, 12H, *J* = 6.8 Hz, Ar-CH(C*H*_3_)_2_), δ = 0.99 (t, 9H, *J* = 7.9 Hz, Ar-SiCH_2_C*H*_3_), δ = 0.78 (q, 6H, *J* = 7.9 Hz, Ar-SiC*H*_2_CH_3_), δ = 0.40 (s, 9H, Cp-Si(C*H*_3_)_3_). ^13^C{^1^H} NMR (125 MHz, CDCl_3_): δ = 165.5, 137.1, 136.8, 133.4, 129.3, 127.9, 122.5 (Aromatic group), δ = 27.0 (Ar-*C*H(CH_3_)_2_), δ = 23.8 (Ar-CH(*C*H_3_)_2_), δ = 7.66 (-SiCH_2_*C*H_3_), δ = 3.74 (-Si*C*H_2_CH_3_), δ = −0.43 (-Si(*C*H_3_)_3_). EA: Found. C 57.18%, H 8.005%; Calcd. for C_26_H_44_C_l2_OSi_2_Ti: C 57.03%, H 8.100%.

Typical Reaction Procedure for Ethylene Copolymerization with Styrene. A typical reaction procedure for the copolymerization of ethylene with styrene by a (Me_3_SiC_5_H_4_)TiCl_2_(O-2,6-*^i^*Pr_2_C_6_H_3_) (**5**)-MAO catalyst (Table 2, run 13) is as follows: Toluene (26.0 mL) and d-MAO (white solid, 174 mg, 3.0 mmol) were added into the autoclave (100 mL, stainless steel) in the dry box, and the reaction apparatus was then filled with ethylene in the fume hood. A toluene solution (1.0 mL) containing **1** was then added into the autoclave after the addition of styrene (3.0 mL), and the reaction apparatus was then immediately pressurized to the prescribed pressure employed. The mixture was stirred for 10 min, and the polymerization was terminated with the addition of MeOH. The solution was then poured into MeOH (100 mL), and the resultant polymer was adequately washed with MeOH and then dried in vacuo for several hours.

According to previous reports [20,21], the resultant polymer mixture was separated into three fractions. Atactic polystyrene prepared by MAO itself (in a cationic manner) was extracted with acetone. Poly(ethylene-*co*-styrene)s were then extracted with THF from the acetone-insoluble portion, and the polyethylene and syndiotactic polystyrene by-products were separated as the THF-insoluble fraction. The basic experimental procedure is as follows: The resultant polymer solids (after precipitation with MeOH and dried in vacuo) were added to acetone (15.0 mL) in a centrifugal sedimentation tube (50 mL, glass), and the copolymers were separated [with the removal of the atactic polystyrene formed due to MAO] as precipitates by a centrifuge. The procedure was repeated three times. The acetone-insoluble fraction was added into a centrifugal sedimentation tube containing tetrahydrofuran (THF, 15.0 mL), and the THF-soluble and THF-insoluble fractions were separated; the procedure was repeated three times. As reported previously [20,21], the resultant copolymers were soluble in THF and the amount of THF insoluble fractions was negligible in all cases. These fractions were analyzed by ^1^H- and ^13^C-NMR spectra, GPC analysis, and DSC thermograms.

## Figures and Tables

**Table 1:** Selected bond distances (Å) and angles (º) in (Me_3_SiC_5_H_4_)TiCl_2_(O-2,6-*^i^*Pr_2_-4-RC_6_H_2_) [R = H (**5**), SiEt_3_ (**6**)], *^a^* Cp*TiCl_2_(O-2,6-*^i^*Pr_2_-4-RC_6_H_2_) [R = H (**1**) [35], SiEt_3_ (**2**) [32]], and CpTiCl_2_(O-2,6-*^i^*Pr_2_C_6_H_3_) (**7**) [35] *^a^*.

	1 *^b^*	2 *^c^*	5	6	7 *^b^*
Bond Distances (Å)					
Ti(1)–Cl(1)	2.305(2)	2.2723(9)	2.2660(5)	2.2721(15)	2.262(1)
Ti(1)–Cl(2)	2.239(2)	2.2677(8)	2.2730(5)	2.2534(14)	2.262(1)
Ti(1)–O(1)	1.772(3)	1.7965(19)	1.7829(13)	1.773(2)	1.760(4)
Si(1)–C(1)	--	1.875(3)	1.876(2)	1.864(7)	--
O(1)–C(6)	1.367(5)	1.363(3)	1.373(2)	1.375(4)	1.368(4)
Ti(1)–C(1)	2.365(7)	2.403(3)	2.3678(17)	2.373(13)	2.282(8)
Ti(1)–C(2)	2.438(7)	2.411(3)	2.3854(19)	2.323(9)	2.299(5)
Ti(1)–C(3)	2.365(7)	2.359(3)	2.348(2)	2.325(8)	2.235(5)
Ti(1)–C(4)	2.311(7)	2.345(3)	2.335(2)	2.390(6)	2.235(5)
Ti(1)–C(5)	2.317(4)	2.357(3)	2.330(2)	2.398(8)	2.299(5)
Bond Angles (°)					
Cl(1)–Ti(1)–Cl(2)	103.45(5)	102.33(3)	105.11(2)	103.74(5)	104.23(7)
Cl(1)–Ti(1)–O(1)	99.1(2)	100.97(7)	102.92(4)	102.31(10)	102.53(9)
Cl(2)–Ti(1)–O(1)	104.1(2)	103.24(7)	102.52(4)	103.04(9)	102.53(9)
Ti(1)–O(1)–C(6)	173.0(3)	174.62(19)	151.41(12)	157.0(2)	163.0(4)

*^a^* CCDC2378274 (complex **5**) and CCDC2378275 (complex **6**) contain the supplementary crystallographic data in detail. *^b^* Cited from reference 35. *^c^* Cited from reference 32.

**Table 2:** Ethylene copolymerization with styrene (St) by Cp′TiCl_2_(O-2,6-*^i^*Pr_2_-4-RC_6_H_2_) [Cp’ = Cp*, R = H (**1**), SiEt_3_ (**2**); Cp’ = *^t^*BuC_5_H_4_, R = H (**3**), SiEt_3_ (**4**); Cp’ = Me_3_SiC_5_H_4_, R = H (**5**), SiEt_3_ (**6**)]–MAO catalyst systems (ethylene 4 atm, 20 °C, 10 min) *^a^*.

Run	cat.	St	Yield *^b^*	Activity *^b^*	THF Soluble Fraction				
		/mL	/mg		cont. *^c^*/wt.%	*M*_n_ *^d^* × 10^−4^	*M*_w_/*M*_n_ *^d^*	*T*_g_ *^e^*/°C	St *^f^*/mol%
1	**1**	3	60.0	720	97	16.7	1.66	−18.5	18.8
2	**1**	5	50.6	610	90	13.5	1.32	−6.6	27.5
3	**1**	10	39.5	470	86	6.86	1.26	15.0	42.6
4	**2**	3	32.4	390	96	9.23	1.65	−15.3	20.6
5	**2**	5	23.5	280	95	9.91	1.41	−10.8	25.4
6	**2**	10	19.2	230	93	7.38	1.14	10.7	40.3
7	**3**	3	429	5150	>99	4.65	1.51	6.6	32.3
8	**3**	5	406	4870	>99	3.98	1.48	27.6	40.5
9	**3**	10	371	4450	>99	3.34	1.87	31.0	51.1
10	**4**	3	197	2360	>99	14.7	1.81	11.4	32.3
11	**4**	5	180	2160	>99	6.76	1.51	22.2	38.1
12	**4**	10	154	1850	>99	5.28	2.05	29.5	46.8
13	**5**	3	323	3880	>99	5.18	1.58	14.2	35.0
14	**5**	5	450	5400	>99	4.65	1.76	23.2	41.3
15	**5**	10	723	8680	>99	3.82	1.70	38.9	54.2
16	**6**	3	361	4330	>99	10.9	1.80	15.3	36.6
17	**6**	5	411	4930	98	12.8	1.79	30.9	44.9
18	**6**	10	506	6070	96	8.78	1.71	41.3	55.6

*^a^* Reaction conditions: catalyst 0.50 µmol, toluene and styrene total 30.0 mL, d-MAO 3.0 mmol, 20 °C, ethylene 4 atm, 10min. *^b^* Activity in kg polymer/mol-Ti∙h, polymer yield in acetone insoluble and THF soluble fraction. *^c^* Percentage (wt%) in acetone insoluble and THF soluble fraction (after removal of atactic polystyrene). *^d^* GPC data in *o*-dichlorobenzene vs. polystyrene standards. *^e^* Glass transition temperature (*T*_g_) measured by DSC thermograms. *^f^* Styrene content in mol% estimated by ^1^H NMR spectra in 1,1,2,2-tetrachloroethane-*d_2_* at 110 °C.

**Table 3:** Ethylene (E) copolymerization with styrene (St) by (Me_3_SiC_5_H_4_)TiCl_2_(O-2,6-*^i^*Pr_2_-4-RC_6_H_2_) [R = H (**5**), SiEt_3_ (**6**)]–MAO catalyst systems: effect of ethylene pressure, styrene concentration, and temperature *^a^*.

Run	cat.	E	St	temp.	Yield *^b^*	Activity *^b^*	THF Soluble Fraction				
	(μmol)	/atm	/mL	/°C	/mg		cont. *^c^*/wt.%	*M*_n_ *^d^* × 10^−4^	*M*_w_/*M*_n_ *^d^*	*T*_g_ *^e^/*°C	St *^f^*/mol%
19	**5** (0.50)	6	3	20	556	6670	97	9.12	1.88	2.0	29.9
13	**5** (0.50)	4	3	20	323	3880	99	5.18	1.58	14.2	35.0
14	**5** (0.50)	4	5	20	450	5400	99	4.65	1.76	23.2	46.6
15	**5** (0.50)	4	10	20	723	8680	>99	3.82	1.70	38.9	54.2
20	**5** (0.30)	4	3	20	192	3840	>99	5.35	1.76	11.4	34.7
19	**5** (0.50)	6	3	20	556	6670	97	9.12	1.88	2.0	29.9
21	**5** (0.30)	4	5	20	223	4460	99	4.78	1.68	25.7	41.0
22	**5** (0.30)	4	10	20	264	5280	>99	4.04	1.80	40.2	54.3
23	**5** (0.50)	4	3	50	162	1940	97	2.24	1.61	18.1	38.9
24 *^g^*	**5** (0.50)	4	3	50	110	1320	95	1.96	1.43	14.7	39.4
25 *^h^*	**5** (0.50)	4	3	50	185	2220	97	2.48	1.43	17.4	40.6
26	**5** (0.50)	4	5	50	199	2390	90	1.46	1.70	29.3	50.0
27	**5** (0.50)	4	10	50	229	2750	86	1.08	1.63	40.6	56.5
28	**5** (0.50)	2	3	20	177	2120	>99	4.45	1.47	33.0	52.6
29	**5** (0.50)	2	10	20	243	2920	98	1.17	1.90	48.5	63.6
30	**6** (0.50)	6	3	20	349	7000	99	20.2	1.99	2.6	27.2
16	**6** (0.50)	4	3	20	361	4330	>99	10.9	1.80	15.3	36.6
17	**6** (0.50)	4	5	20	411	4930	98	12.8	1.79	30.9	44.9
18	**6** (0.50)	4	10	20	506	6070	96	8.78	1.71	41.3	55.6
31	**6** (0.50)	4	3	50	217	2610	96	3.84	1.64	18.1	38.8
32	**6** (0.50)	4	5	50	291	3490	96	3.18	2.04	32.7	46.6
33	**6** (0.50)	4	10	50	359	4310	83	2.95	2.04	44.7	55.1

*^a^* Reaction conditions: toluene and styrene total 30.0 mL, d-MAO 3.0 mmol, 10 min. *^b^* Activity in kg polymer/mol-Ti∙h, polymer yield in acetone insoluble and THF soluble fraction. *^c^* Percentage (wt%) in acetone insoluble and THF soluble fraction (after removal of atactic polystyrene). *^d^* GPC data in *o*-dichlorobenzene vs. polystyrene standards. *^e^* Glass transition temperature (*T*_g_) measured by DSC thermograms. *^f^* Styrene content in mol% estimated by ^1^H NMR spectra in 1,1,2,2-tetrachloroethane-*d_2_* at 110 °C. *^g^* MAO 1.0 mmol. *^h^* MAO 2.0 mmol.

## Data Availability

The data are contained within the article and the Supplementary Material. The crystallographic analysis data for (Me_3_SiC_5_H_4_)TiCl_2_(O-2,6-*^i^*Pr_2_-4-RC_6_H_2_) [R = H (**5**), SiEt_3_ (**6**)] are available as CCDC 2378274 and 2378275, respectively, from The Cambridge Crystallographic Data Centre (CCDC).

## Supplementary Materials

The following supporting information can be downloaded at: https://www.mdpi.com/article/10.3390/molecules29184473/s1, Crystal data and collection parameters for analysis of (Me_3_SiC_5_H_4_)TiCl_2_(O-2,6-*^i^*Pr_2_-4-RC_6_H_2_) [R = H (**5**), SiEt_3_ (**6**)]; ethylene polymerization data for **1**–**4**; selected NMR spectra of poly(ethylene-*co*-styrene)s and selected DSC thermograms in resultant poly(ethylene-*co*-styrene)s.
