# Supplementary material for: Ethylene/Styrene Copolymerization by (Me3SiC5H4)TiCl2(O-2,6-iPr2-4-RC6H2) (R = H, SiEt3)-MAO Catalysts: Effect of SiMe3 Group on Cp for Efficient Styrene Incorporation
Source: Molecules. 2024 Sep 20;29(18):4473. doi: 10.3390/molecules29184473 (PMC11434206; doi:10.3390/molecules29184473)
Supplement: Supplementary file 1 [file molecules-29-04473-s001.zip › molecules-3198260-supplementary.pdf]

Supplementary Materials

for

Ethylene/Styrene Copolymerization by $(Me_3SiC_5H_4)TiCl_2(O\text{-}2,6\text{-}{}^iPr_2\text{-}4\text{-}RC_6H_2)$ (R = H, $SiEt_3$)-MAO Catalysts: Effect of $SiMe_3$ group on Cp for Efficient Styrene Incorporation

Tiantian Huang, Taiga Fujioka, Daisuke Shimoyama and Kotohiro Nomura*

Department of Chemistry, Tokyo Metropolitan University, 1-1 Minami Osawa, Hachioji, Tokyo 192-0397, Japan; ktnomura@tmu.ac.jp

*Correspondence: ktnomura@tmu.ac.jp

Contents

		page
1	Crystal data and the collection parameters for analysis of $(Me_3SiC_5H_4)TiCl_2(O\text{-}2,6\text{-}{}^iPr_2\text{-}4\text{-}RC_6H_2)$ [R = H (**5**), $SiEt_3$ (**6**)]	S2
2	Results in ethylene polymerization	S3
3	Selected NMR spectra of poly(ethylene-*co*-styrene)s	S4-13
4	3. Selected DSC themongram in the resultant poly(ethylene-*co*-styrene)s.	S14

1. Crystal data and the collection parameters for analysis of ($(Me_3SiC_5H_4)TiCl_2(O\text{-}2,6\text{-}{}^iPr_2\text{-}4\text{-}RC_6H_2)$) [R = H (5), $SiEt_3$ (6)].

Table S1. Crystal data and the collection parameters for analysis of $(Me_3SiC_5H_4)TiCl_2(O\text{-}2,6\text{-}{}^iPr_2\text{-}4\text{-}RC_6H_2)$ [R = H (**5**), $SiEt_3$ (**6**)].[a]

	**5**	**6**
formula	$C_{20}H_{30}Cl_2OSiTi$	$C_{26}H_{44}Cl_2OSi_2Ti$
formula weight	433.30	547.56
crystal color, Habit	orange, plate	orange, plate
crystal size (mm)	0.388×0.178×0.040	0.200×0.140×0.023
crystal system	monoclinic	monoclinic
space group	$P1\ 2_1/n\ 1$	$P2_1/c$
a (Å)	6.6649(3)	22.5185(10)
b (Å)	11.8405(5)	11.0402(4)
c (Å)	28.8139(11)	12.3590(5)
α (deg)		
β (deg)	94.699(4)	99.407(4)
γ (deg)		
V (Å^3)	2266.23(16)	3031.2(2)
Z value	4	4
D_{calcd} (g/cm^3)	1.270	1.200
F_{000}	912	1168.0
temp (K)	123(2)	123(2)
μ (Mo Kα) (cm^{-1})	6.72	5.54
no. of reflections measured (R_{int})	17719	42534
	Unique:5515 (0.0845)	Unique: 7179 (0.0651)
$2\theta_{max}$ (deg)	56.2	55.6
no. of observations [$I > 2.00\sigma(I)$]	5515	7179
no. of variables	233	397
$R1$ [$I > 2.00\sigma(I)$]	0.0434	0.0685,
$wR2$ [$I > 2.00\sigma(I)$]	0.1146	0.1238
goodness of fit	1.045	1.091

[a] CCDC2378274 (complex **5**), CCDC2378275 (complex **6**)

2. Restkts in ethylene polymerization

Table S2. Ethylene polymerization by $Cp'TiCl_2(O\text{-}2,6\text{-}{}^iPr_2\text{-}4\text{-}RC_6H_2)$ [Cp' = Cp*, R = H (**1**), $SiEt_3$ (**2**); Cp' = ${}^tBuC_5H_4$, R = H (**3**), $SiEt_3$ (**4**)]–MAO catalyst systems (toluene, 10 min). [a]

catalyst (μmol)	ethylene / atm	temp. / °C	yield / mg	activity[b] kg-PE/mol-Ti·h	$M_n{}^c\times10^{-4}$	$M_w/M_n{}^c$
**1** (0.1)	6	20	283	17000	371	1.86
**1** (0.015)[d]	4	25	119	47400		
**2** (0.05)	6	20	207	24900	195	1.37
**2** (0.05)	4	20	159	19200	155	1.58
**2** (0.015)[d]	4	25	142	56800		
**3** (0.2)	6	20	165	4950	72.1	2.42
**4** (0.2)	6	20	332	9970	76.8	1.59
**4** (0.2)	4	20	180	5410	69.7	1.81
**1** (0.1)	6	50	247	14800		
**2** (0.05)	6	50	140	16900	106	1.69
**3** (0.2)	6	50	129	3870	44.1	3.02
**4** (0.2)	6	50	172	5160	25.1	2.31
**4** (0.2)	4	50	103	3090	25.1	3.02

[a]Conditions: toluene and catalyst solution total 30.0 mL and 10 min, MAO 3.0 mmol. [b]Activity in kg-PE/mol-Ti·h. [c] Gel permeation chromatography (GPC) data in *o*-dichlorobenzene vs polystyrene standards. [d]Cited from Kitphaitun, S.; Yan, Q.; Nomura, K. *Angew. Chem. Int. Ed.* **2020**, *59*, 23072-23076.

3. Selected NMR spectra of poly(ethylene-*co*-styrene)s.

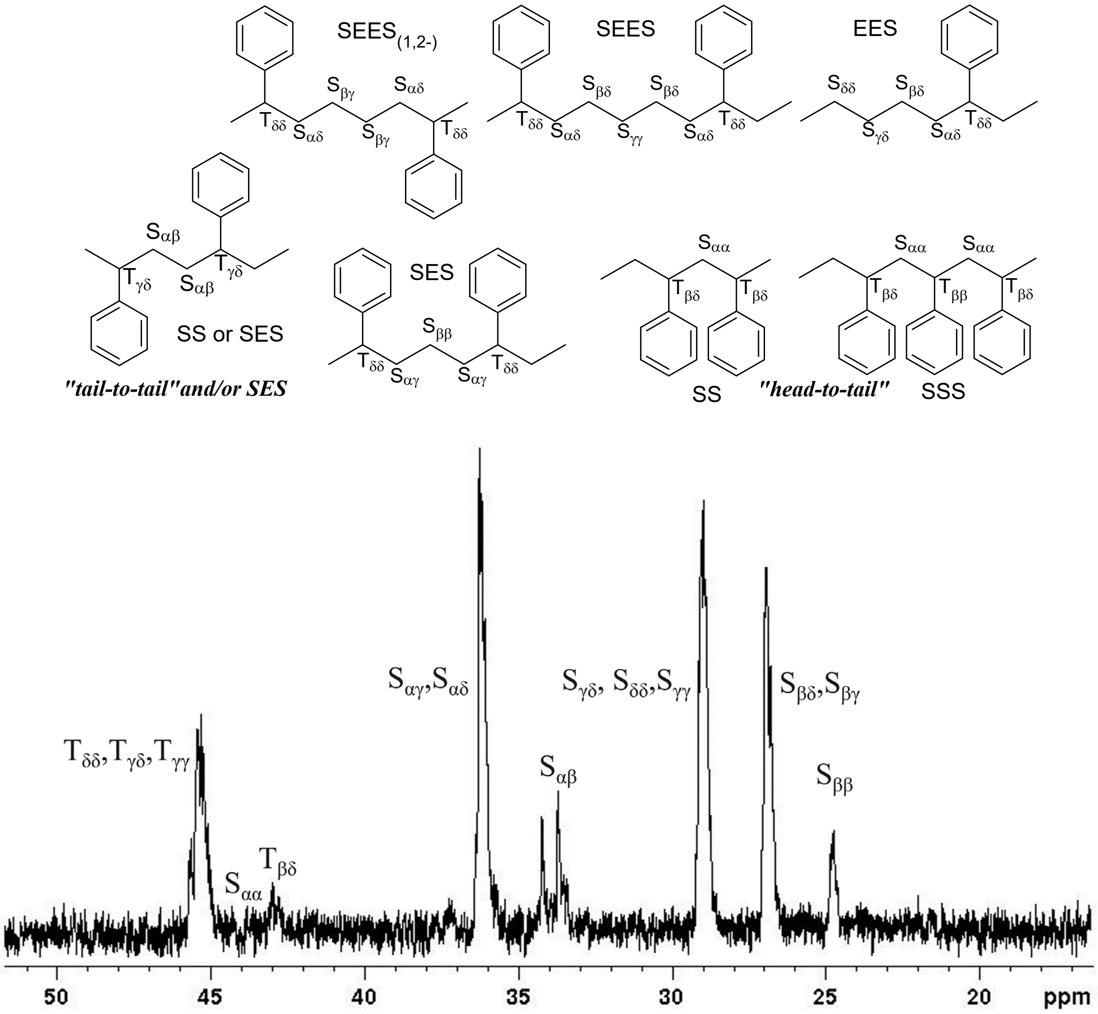

Figure S1. ^{13}C NMR spectrum (in 1,1,2,2-tetrachloroethane-d_2 solution at 110 °C) for poly(ethylene-*co*-styrene) prepared by $(Me_3SiC_5H_4)TiCl_2(O\text{-}2,6\text{-}^{i}Pr_2C_6H_3)$ (**5**)–MAO catalyst system, styrene content 35.0 mol % (run 13).

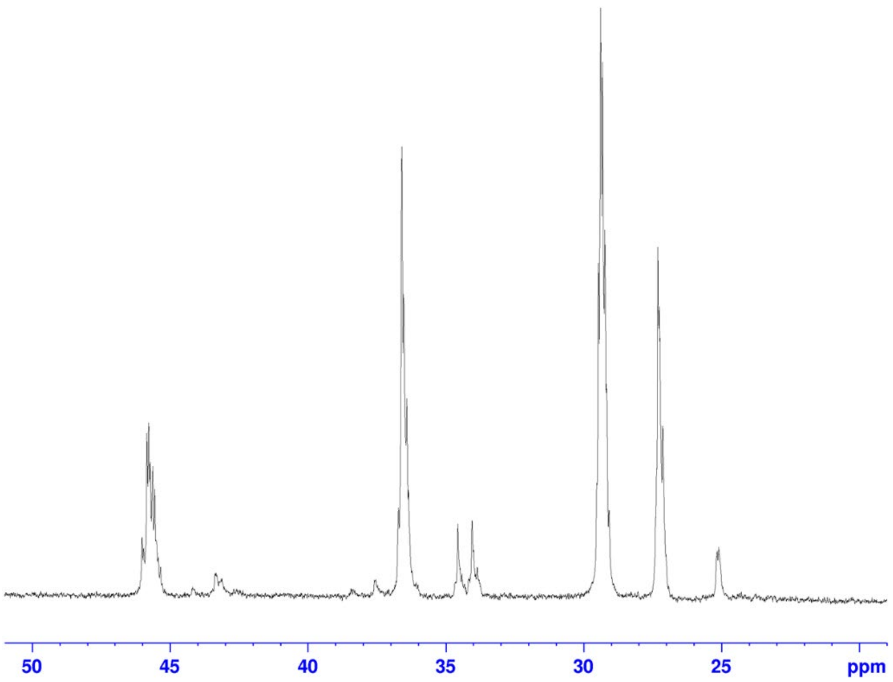

Figure S2. ^{13}C NMR spectrum (in 1,1,2,2-tetrachloroethane-d_2 solution at 110 °C) for poly(ethylene-*co*-styrene) prepared by $(Me_3SiC_5H_4)TiCl_2(O\text{-}2,6\text{-}{}^iPr_2C_6H_3)$ (**5**)–MAO catalyst system, styrene content 29.9 mol % (run 19).

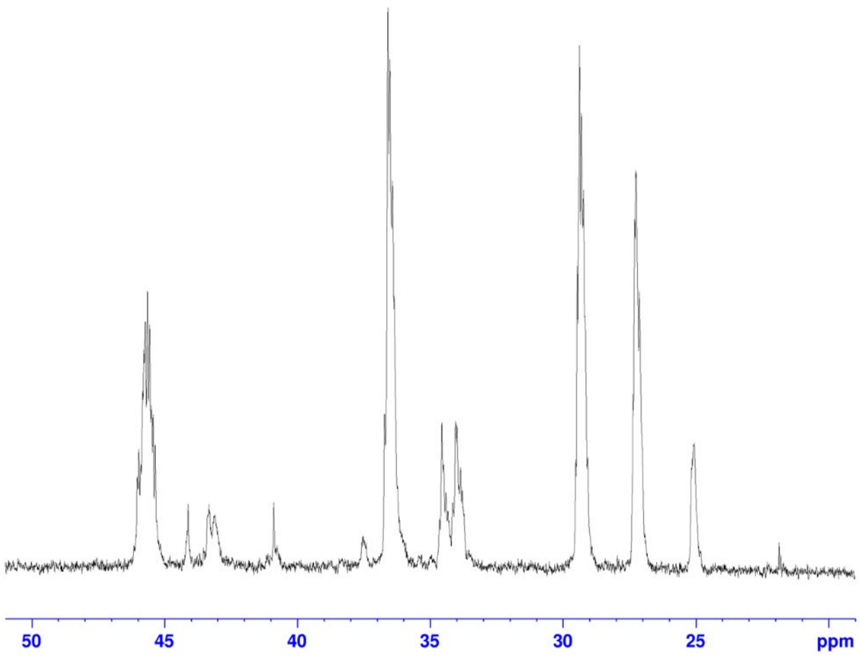

Figure S3. ^{13}C NMR spectrum (in 1,1,2,2-tetrachloroethane-d_2 solution at 110 °C) for poly(ethylene-*co*-styrene) prepared by $(Me_3SiC_5H_4)TiCl_2(O\text{-}2,6\text{-}{}^iPr_2C_6H_3)$ (**5**)–MAO catalyst system, styrene content 38.9 mol % (run 23).

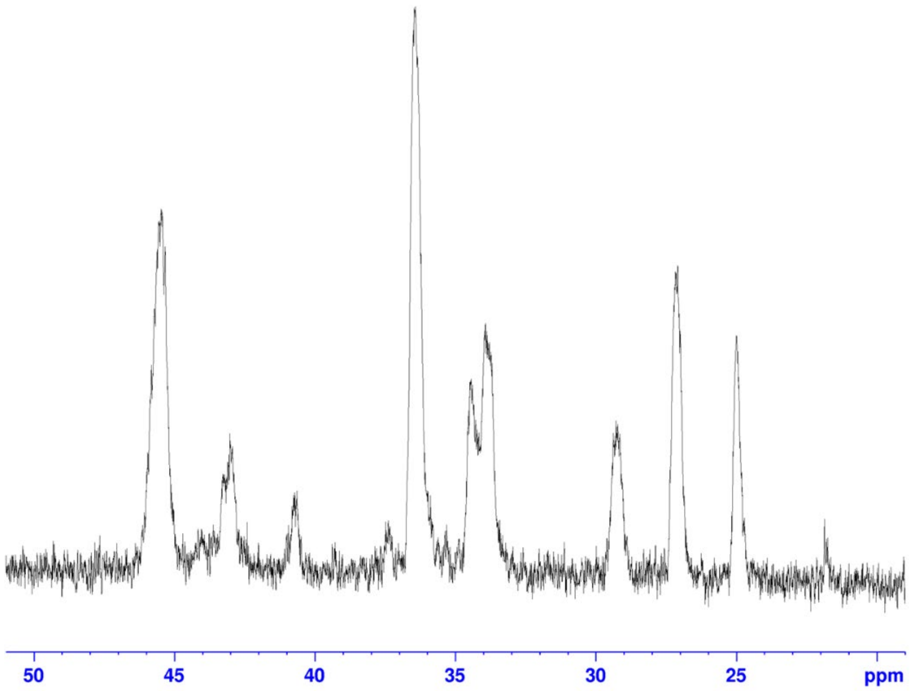

Figure S4. ^{13}C NMR spectrum (in 1,1,2,2-tetrachloroethane-d_2 solution at 110 °C) for poly(ethylene-*co*-styrene) prepared by $(Me_3SiC_5H_4)TiCl_2(O\text{-}2,6\text{-}^iPr_2C_6H_3)$ (**5**)–MAO catalyst system, styrene content 54.2 mol % (run 15).

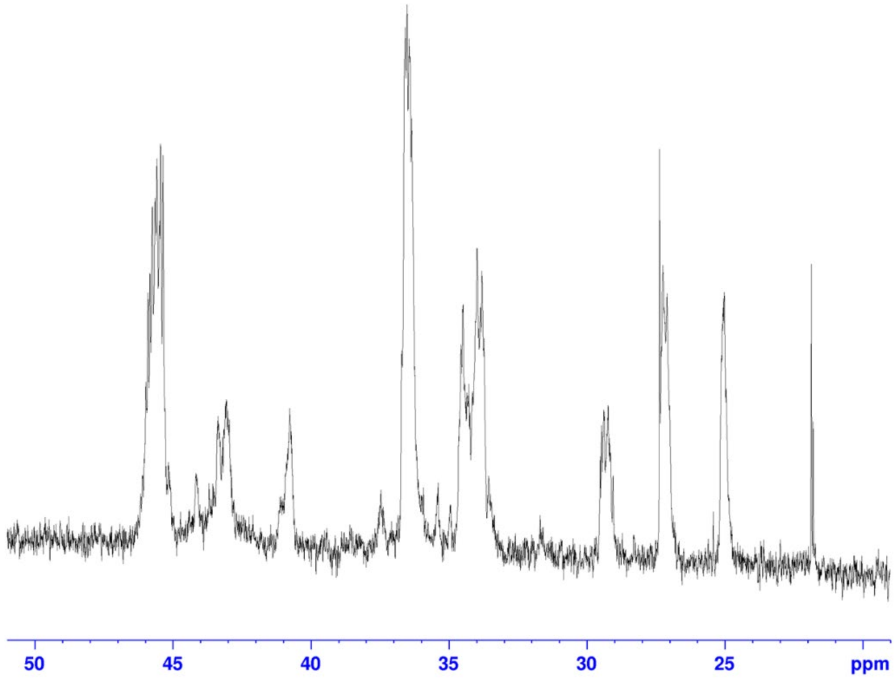

Figure S5. ^{13}C NMR spectrum (in 1,1,2,2-tetrachloroethane-d_2 solution at 110 °C) for poly(ethylene-*co*-styrene) prepared by $(Me_3SiC_5H_4)TiCl_2(O\text{-}2,6\text{-}^iPr_2C_6H_3)$ (**5**)–MAO catalyst system, styrene content 56.5 mol % (run 27).

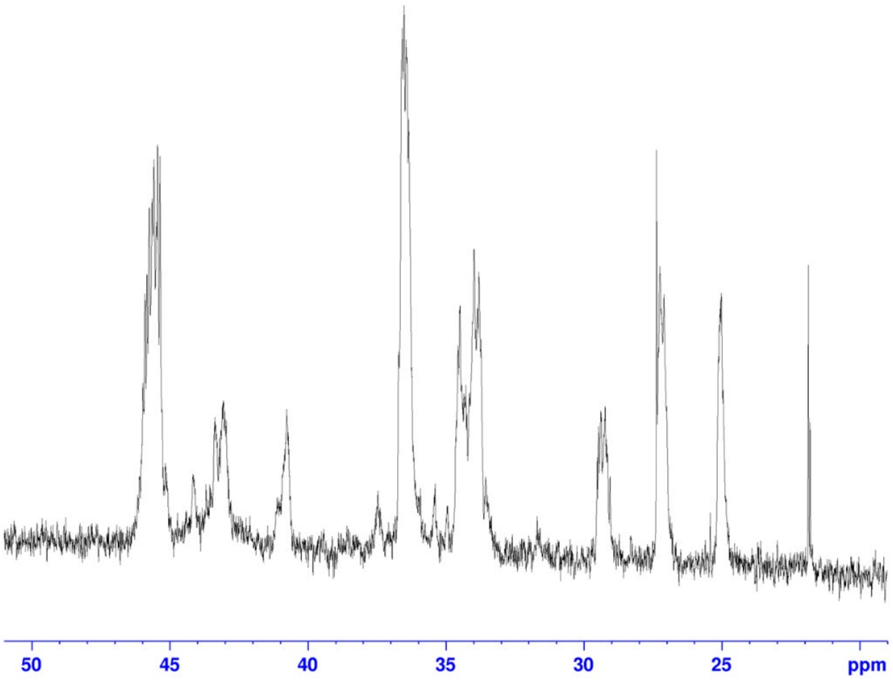

Figure S6. ^{13}C NMR spectrum (in 1,1,2,2-tetrachloroethane-d_2 solution at 110 °C) for poly(ethylene-*co*-styrene) prepared by ($Me_3SiC_5H_4$)$TiCl_2$(O-2,6-^{i}Pr_2-4-$SiEt_3C_6H_2$) (**6**)–MAO catalyst system, styrene content 36.6 mol % (run 16).

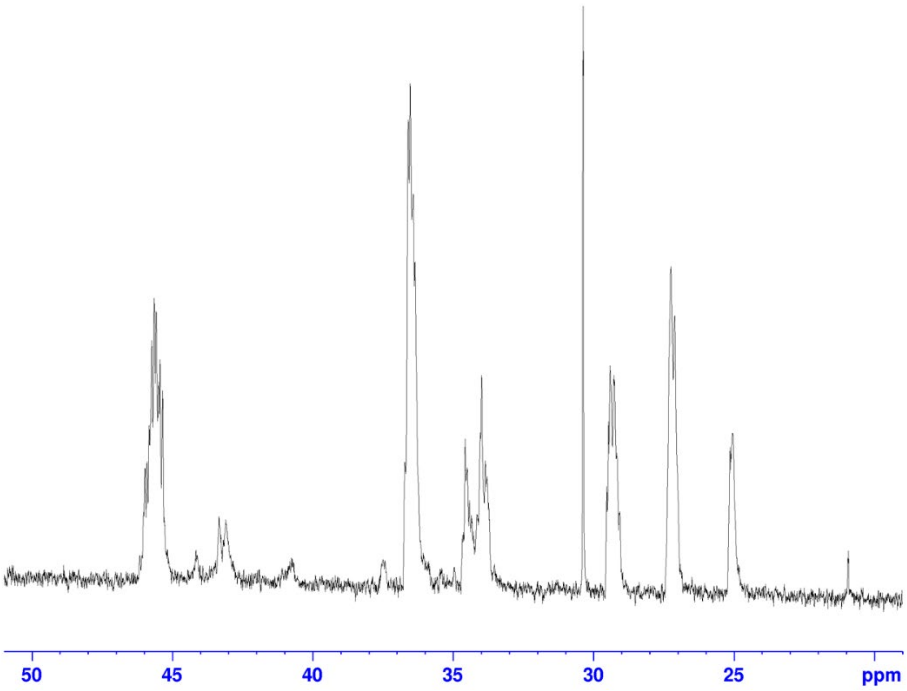

Figure S7. ^{13}C NMR spectrum (in 1,1,2,2-tetrachloroethane-d_2 solution at 110 °C) for poly(ethylene-*co*-styrene) prepared by ($Me_3SiC_5H_4$)$TiCl_2$(O-2,6-^{i}Pr_2-4-$SiEt_3C_6H_2$) (**6**)–MAO catalyst system, styrene content 44.9 mol % (run 17).

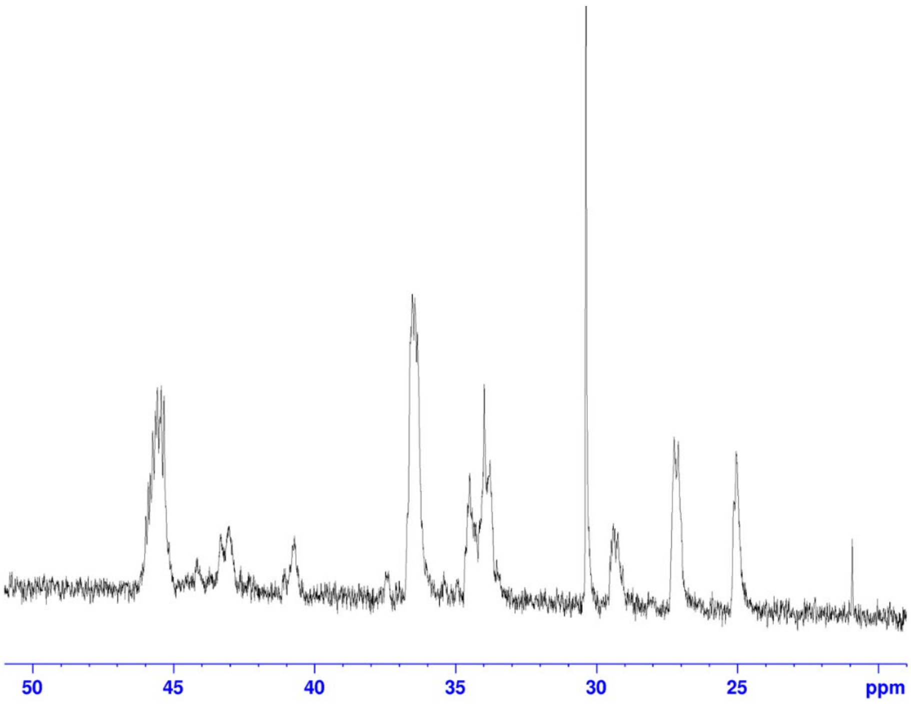

Figure S8. ^{13}C NMR spectrum (in 1,1,2,2-tetrachloroethane-d_2 solution at 110 °C) for poly(ethylene-*co*-styrene) prepared by ($Me_3SiC_5H_4$)$TiCl_2$(O-2,6-$^iPr_2$-4-$SiEt_3C_6H_2$) (**6**)–MAO catalyst system, styrene content 55.6 mol % (run 18).

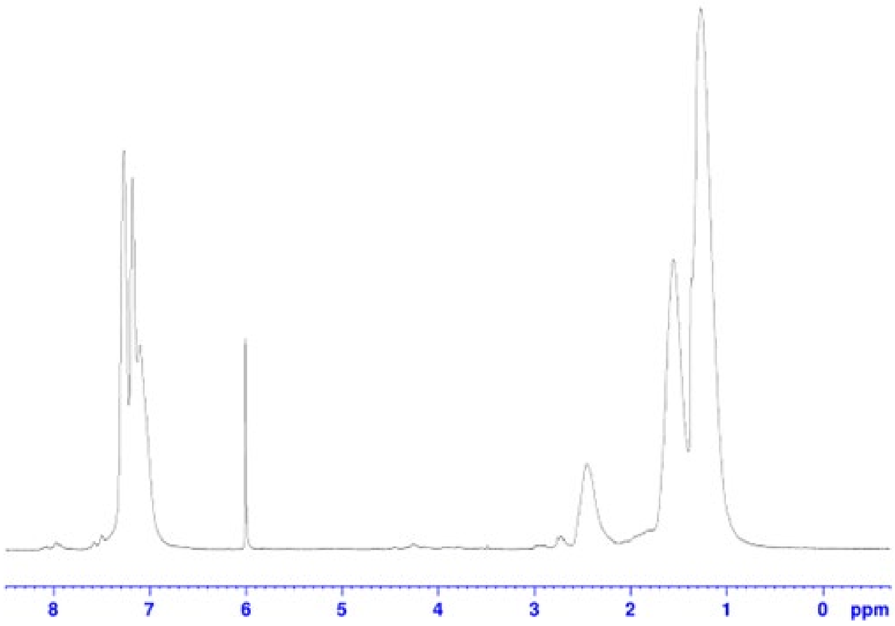

Figure S9. ^{1}H NMR spectrum (in 1,1,2,2-tetrachloroethane-d_2 solution at 110 °C) for poly(ethylene-*co*-styrene) prepared by ($Me_3SiC_5H_4$)$TiCl_2$(O-2,6-$^iPr_2C_6H_3$) (**5**)–MAO catalyst system, styrene content 29.9 mol % (run 19).

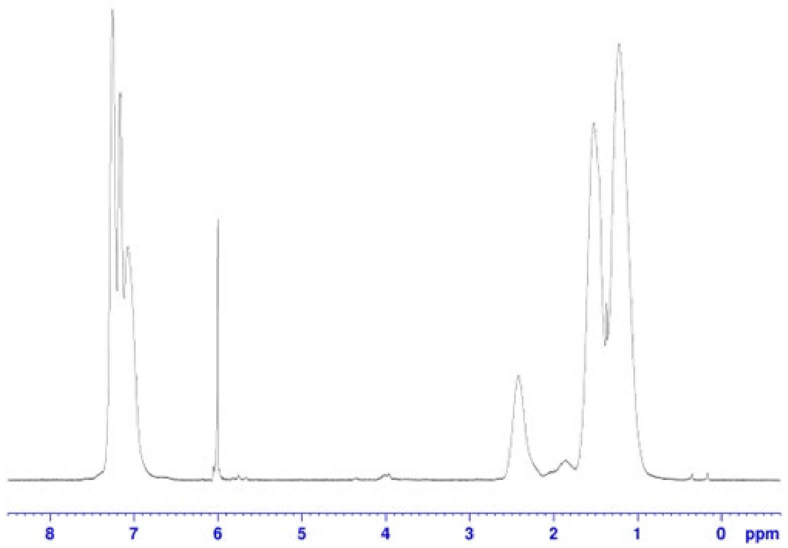

Figure S10. ^{1}H NMR spectrum (in 1,1,2,2-tetrachloroethane-d_2 solution at 110 °C) for poly(ethylene-*co*-styrene) prepared by $(Me_3SiC_5H_4)TiCl_2(O\text{-}2,6\text{-}{}^iPr_2C_6H_3)$ (**5**)–MAO catalyst system, styrene content 35.0 mol % (run 13).

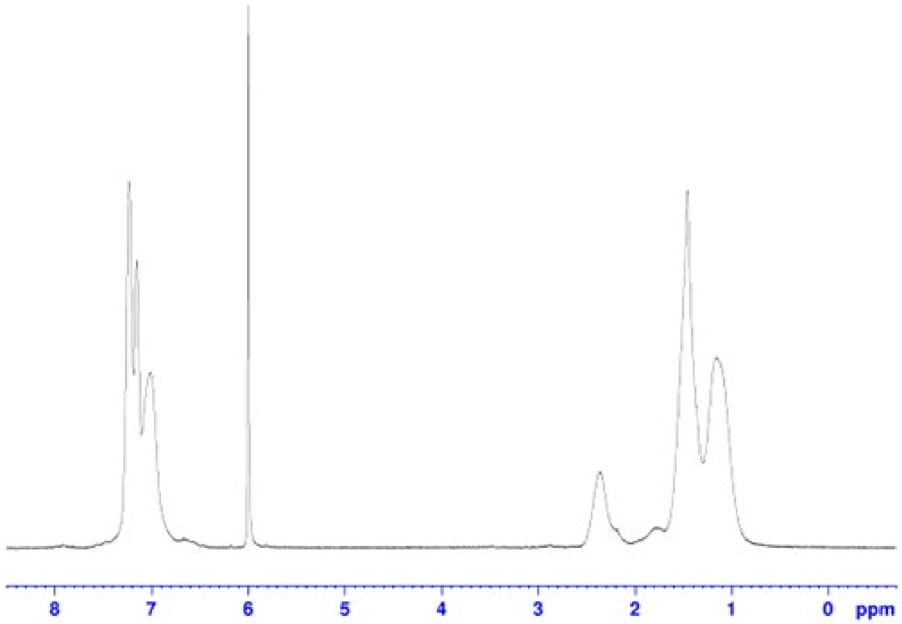

Figure S11. ^{1}H NMR spectrum (in 1,1,2,2-tetrachloroethane-d_2 solution at 110 °C) for poly(ethylene-*co*-styrene) prepared by $(Me_3SiC_5H_4)TiCl_2(O\text{-}2,6\text{-}{}^iPr_2C_6H_3)$ (**5**)–MAO catalyst system, styrene content 41.0 mol % (run 21).

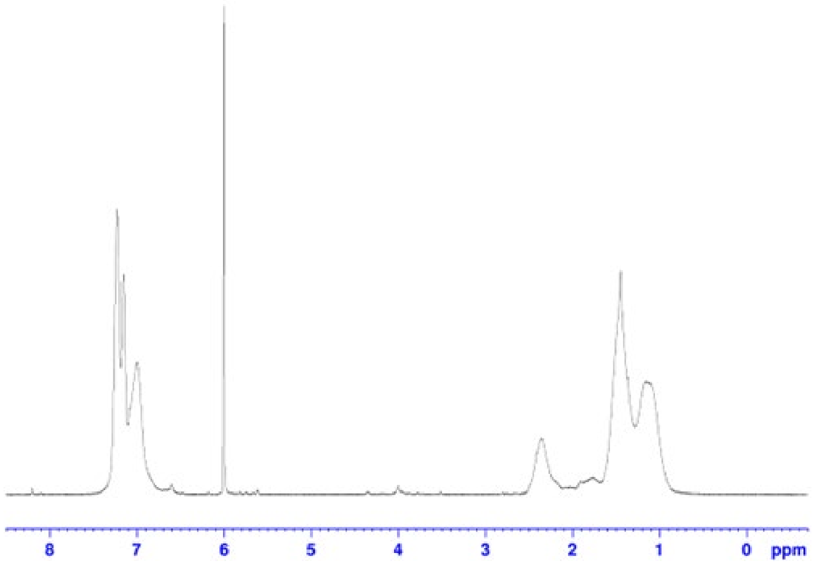

Figure S12. ^{1}H NMR spectrum (in 1,1,2,2-tetrachloroethane-d_2 solution at 110 °C) for poly(ethylene-*co*-styrene) prepared by $(Me_3SiC_5H_4)TiCl_2(O\text{-}2,6\text{-}{}^iPr_2C_6H_3)$ (**5**)–MAO catalyst system, styrene content 50.0 mol % (run 26).

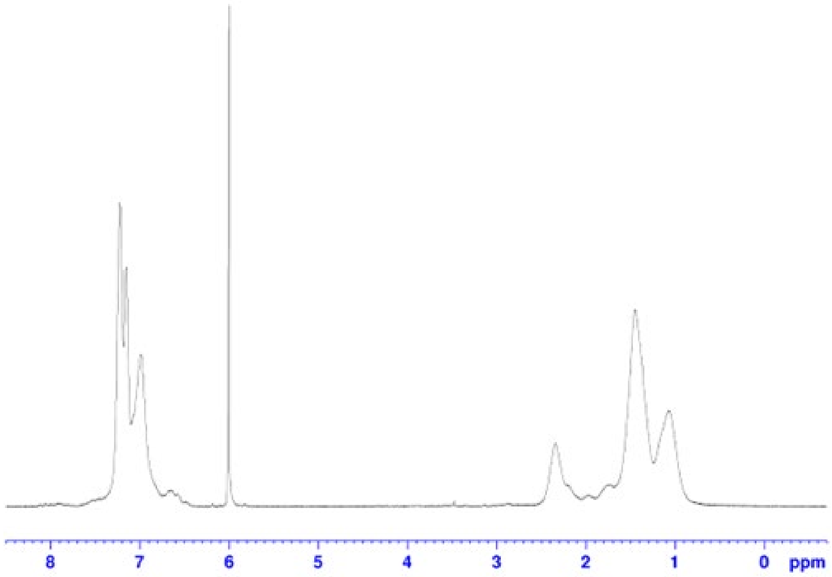

Figure S13. ^{1}H NMR spectrum (in 1,1,2,2-tetrachloroethane-d_2 solution at 110 °C) for poly(ethylene-*co*-styrene) prepared by $(Me_3SiC_5H_4)TiCl_2(O\text{-}2,6\text{-}{}^iPr_2C_6H_3)$ (**5**)–MAO catalyst system, styrene content 54.3 mol % (run 22).

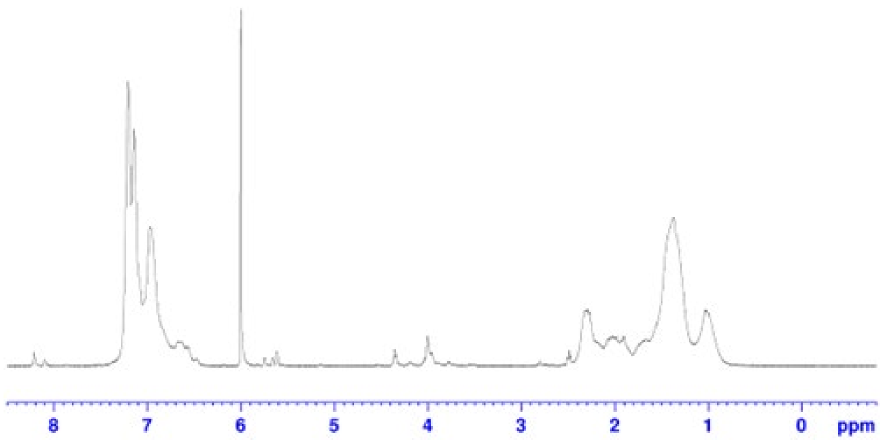

Figure S14. ^{1}H NMR spectrum (in 1,1,2,2-tetrachloroethane-d_2 solution at 110 °C) for poly(ethylene-*co*-styrene) prepared by $(Me_3SiC_5H_4)TiCl_2(O\text{-}2,6\text{-}{}^iPr_2C_6H_3)$ (**5**)–MAO catalyst system, styrene content 63.6 mol % (run 29).

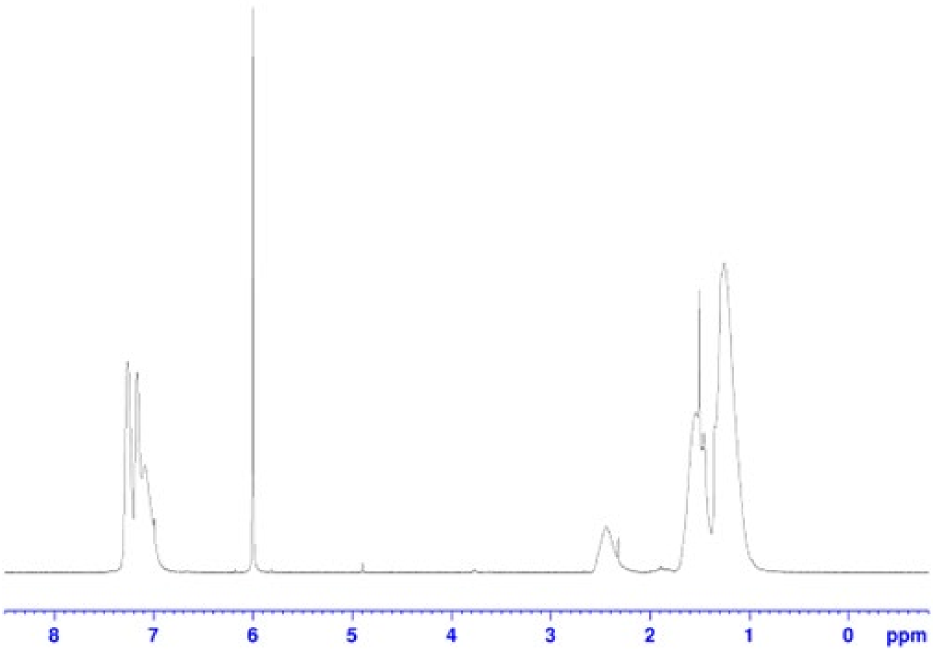

Figure S15. ^{1}H NMR spectrum (in 1,1,2,2-tetrachloroethane-d_2 solution at 110 °C) for poly(ethylene-*co*-styrene) prepared by $(Me_3SiC_5H_4)TiCl_2(O\text{-}2,6\text{-}{}^iPr_2\text{-}4\text{-}SiEt_3C_6H_2)$ (**6**)–MAO catalyst system, styrene content 27.2 mol % (run 30).

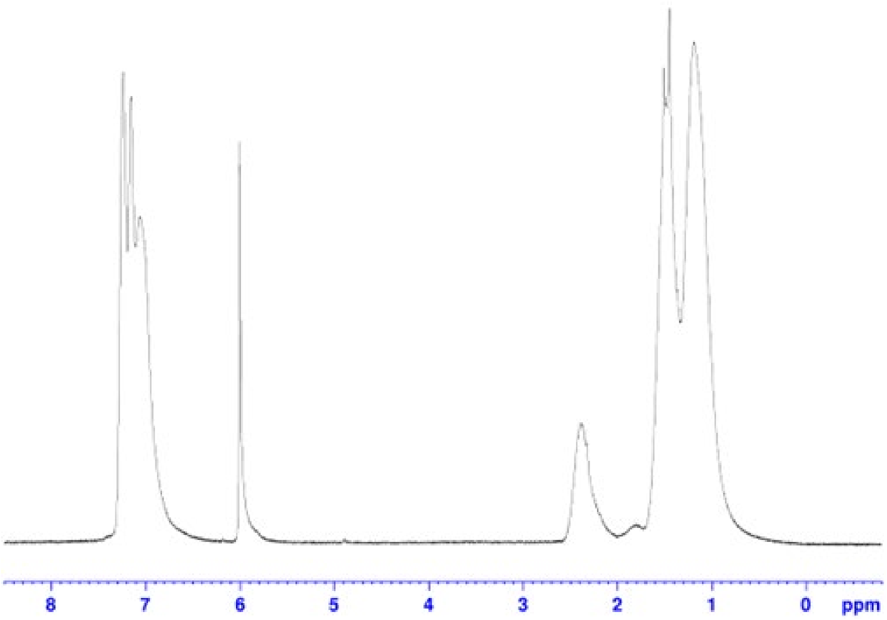

Figure S16. ^{1}H NMR spectrum (in 1,1,2,2-tetrachloroethane-d_2 solution at 110 °C) for poly(ethylene-*co*-styrene) prepared by $(Me_3SiC_5H_4)TiCl_2(O\text{-}2,6\text{-}^{i}Pr_2\text{-}4\text{-}SiEt_3C_6H_2)$ (**6**)–MAO catalyst system, styrene content 36.6 mol % (run 16).

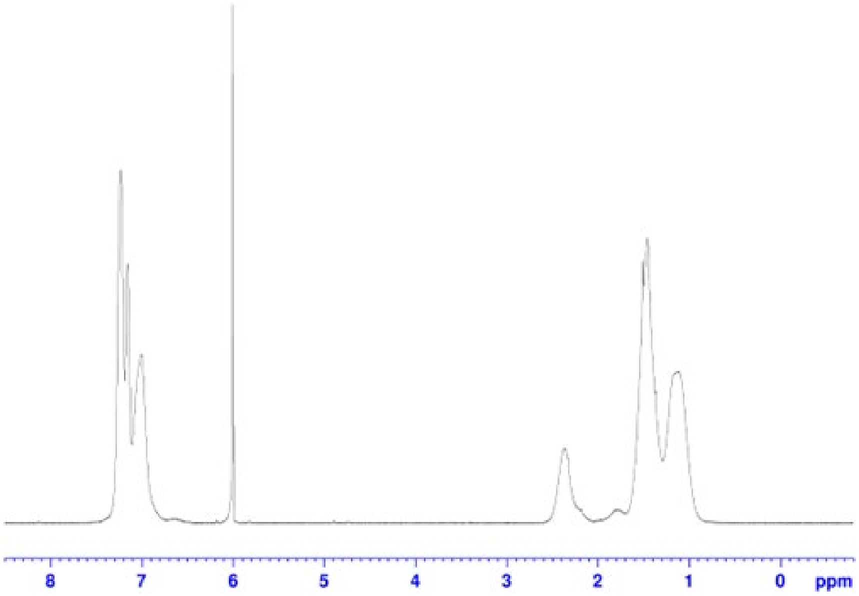

Figure S17. ^{1}H NMR spectrum (in 1,1,2,2-tetrachloroethane-d_2 solution at 110 °C) for poly(ethylene-*co*-styrene) prepared by $(Me_3SiC_5H_4)TiCl_2(O\text{-}2,6\text{-}^{i}Pr_2\text{-}4\text{-}SiEt_3C_6H_2)$ (**6**)–MAO catalyst system, styrene content 44.9 mol % (run 17).

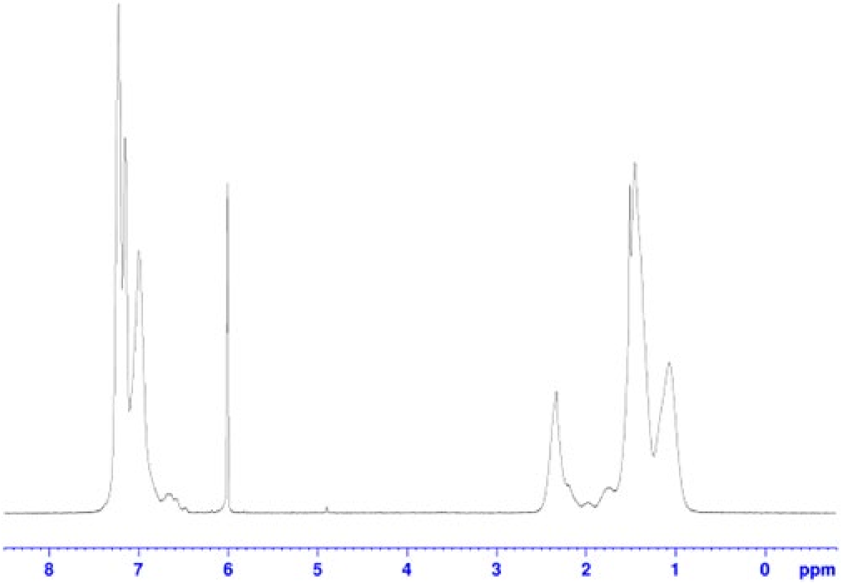

Figure S18. ^{1}H NMR spectrum (in 1,1,2,2-tetrachloroethane-d_2 solution at 110 °C) for poly(ethylene-*co*-styrene) prepared by ($Me_3SiC_5H_4$)$TiCl_2$(O-2,6-$^{i}Pr_2$-4-$SiEt_3C_6H_2$) (**6**)–MAO catalyst system, styrene content 55.6 mol % (run 18).

4. Selected DSC themongram in the resultant poly(ethylene-*co*-styrene)s.

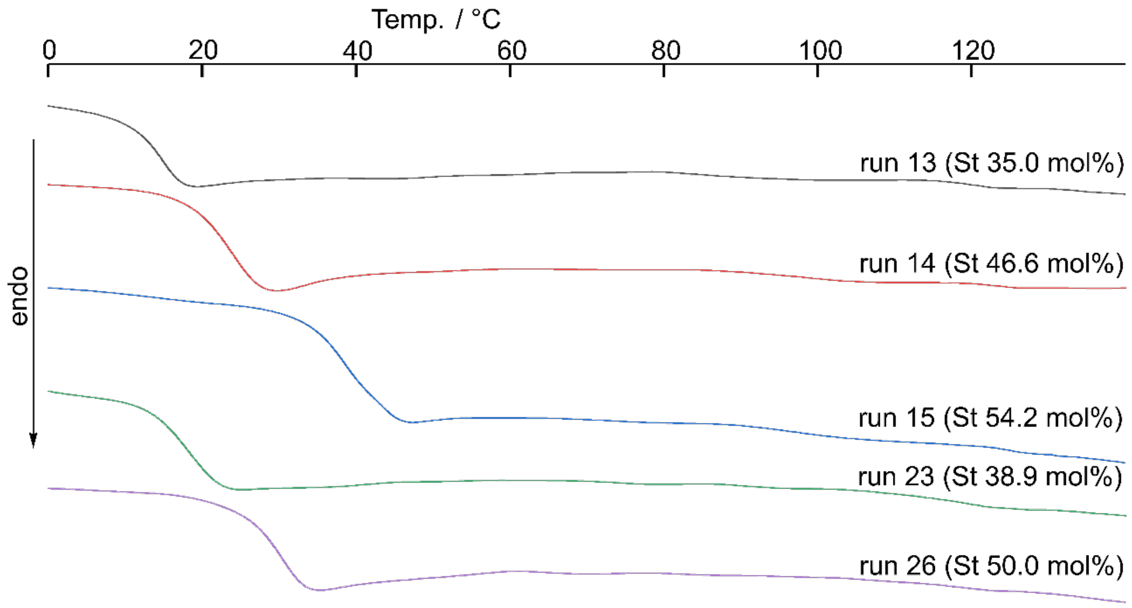

Figure S19. Selected DSC thermograms in the resultant poly(ethylene-*co*-styrene)s prepared by $(Me_3SiC_5H_4)TiCl_2(O\text{-}2,6\text{-}{}^{i}Pr_2C_6H_3)$ (**5**)–MAO catalyst system.

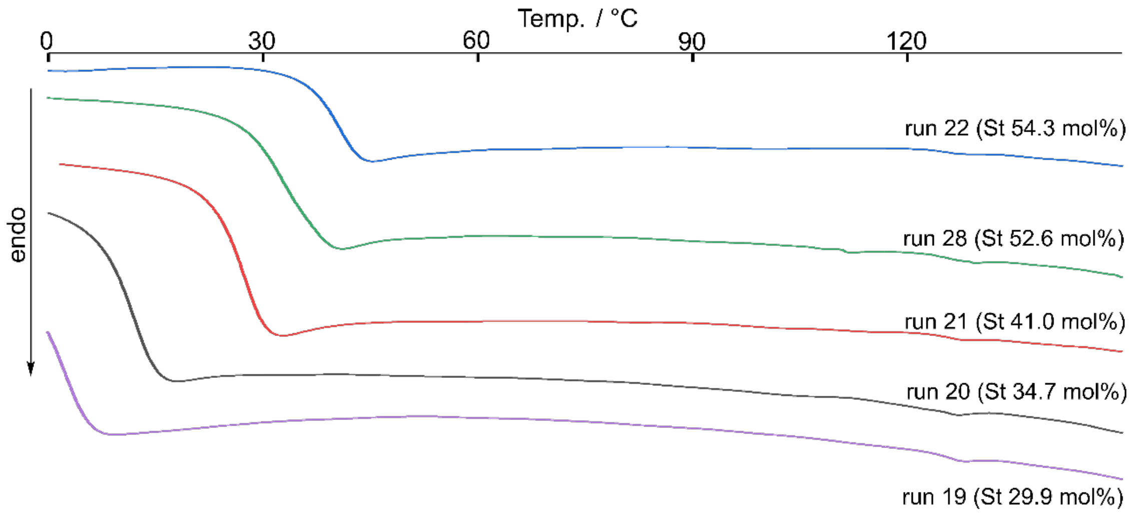

Figure S20. Selected DSC thermograms in the resultant poly(ethylene-*co*-styrene)s prepared by $(Me_3SiC_5H_4)TiCl_2(O\text{-}2,6\text{-}{}^{i}Pr_2C_6H_3)$ (**5**)–MAO catalyst system.
